# Empirical dietary inflammatory pattern and risk of metabolic syndrome and its components: Tehran Lipid and Glucose Study

**DOI:** 10.1186/s13098-019-0411-4

**Published:** 2019-02-12

**Authors:** Zeinab Shakeri, Parvin Mirmiran, Sajjad Khalili-Moghadam, Firoozeh Hosseini-Esfahani, Asal Ataie-Jafari, Fereidoun Azizi

**Affiliations:** 1 Department of Nutrition, Science and Research Branch, Islamic Azad University, Tehran, Iran; 2 Nutrition and Endocrine Research Center, Research Institute for Endocrine Sciences, Shahid Beheshti University of Medical Sciences, Tehran, Iran; 3 Endocrine Research Center, Research Institute for Endocrine Sciences, Shahid Beheshti University of Medical Sciences, Tehran, Iran

**Keywords:** Metabolic syndrome, Empirical dietary inflammatory pattern, Adult

## Abstract

**Background:**

The rising incidence of metabolic syndrome (MetS) is a major public health problem. The inflammatory potential of diet contributes to the development of MetS. The aim of this study was to investigate the relationship between empirical dietary inflammatory pattern (EDIP) and risk of MetS among the Tehranian population. Our hypothesis was that high EDIP would increase the risk of MetS and its components.

**Methods:**

In this prospective study, 2216 adults were selected from among the Tehran Lipid and Glucose Study participants. The usual dietary intakes were estimated using a valid and reliable food frequency questionnaire. Biochemical and anthropometric measurements were assessed at baseline and over 6.2 years of follow up. MetS components were defined according to the modified national Cholesterol Education Program Adult Treatment Panel III. The inflammatory potential of diet was calculated using EDIP score; more positive scores means higher pro-inflammatory diet. Adjusted logistic regression models were used to estimate the occurrence of MetS and its components across quartiles of EDIP score.

**Results:**

Mean ± SD for EDIP score was 0.61 ± 0.40 (range − 2.3 to 6.9). Participants with the highest EDIP scores, had a higher risk of MetS incidence compared to those with the lowest score (OR: 1.75, 95% CI 1.21–2.54, P_trend_ = 0.003). Among the MetS components, hyperglycemia, abdominal obesity, and low HDL-C had a significant positive association with EDIP score; (OR: 1.46, 95% CI 1.03–2.08, P_trend_ = 0.026), (OR: 1.43, 95% CI 1.03–1.97, P_trend_ = 0.046), and (OR: 1.57, 95% CI 1.34–2.19, P_trend_ = 0.015), respectively. No significant association was found between EDIP score, hypertension and hypertriglyceridemia.

**Conclusion:**

Our finding indicated that higher intake of the pro-inflammatory diet may be an independent risk factor for the development of MetS, hyperglycemia, low HDL-C and abdominal obesity in Tehranian adults.

## Introduction

Metabolic syndrome (MetS) is a series of metabolic abnormalities, including central obesity, hypertension, elevated serum triacylglycerol (TG), elevated fasting blood glucose (FBG) and low high-density lipoprotein cholesterol (HDL-C) [1]. Having Mets can increase the risk of chronic diseases including diabetes and cardiovascular diseases (CVD) and their related morbidity and mortalities [2, 3]. Also, high inflammation has been reported in individuals with MetS which could potentially increase CVD incidence [4]. The prevalence of MetS in Iranian adults, has been reported to be more than 30% [5]. Lifestyle components, such as dietary intake, smoking and physical activity, have an important role in the developing of MetS [6]; over the past decade, unhealthy lifestyle behaviors in the Iranian population has led to a high prevalence of MetS [7].

Among lifestyle factors, more emphasis is made on the important role of dietary pattern on the development of MetS [8, 9]. On the other hand, diet is a factor potentially contributing to the development or modulation of inflammation [10, 11]. The inflammatory potential of diet can be determined using the empirical dietary inflammatory pattern (EDIP), which is a hypothesis-driven index that used to evaluate the inflammatory potential of the diet based on the food groups consumption [12].

In previous cross-sectional and cohort studies, no significant association was found between dietary inflammatory index (DII) and MetS [13–15]. In another cohort study with 12.4 years of follow up, high pro-inflammatory diet was associated with increased incidence of MetS [16]. Also, in a cross sectional study, a positive association was revealed between pro-inflammatory diet and waist circumference (WC), as a component of MetS [17]. Some of these cross-sectional studies were unable to determine the causal association between EDII and MetS [13, 14]. Interestingly, a recent meta-analysis demonstrated that higher Dietary Inflammatory Index is associated with higher risk of CVD and CVD-related mortality [18]. Also high inflammation has been reported in individuals with MetS which potentially could increase the CVD incidence [15]. There are limited longitudinal epidemiological studies on this topic; in addition, some studies were mostly limited to assessment of the food parameters of DII [16]. Our hypothesis was that high EDIP would increase the risk of MetS and its components. Hence, the purpose of the present longitudinal study was to examine EDIP score in relation to the risk of MetS and its components, in adult participants.

## Materials and methods

### Study population

**Fig. 1:**
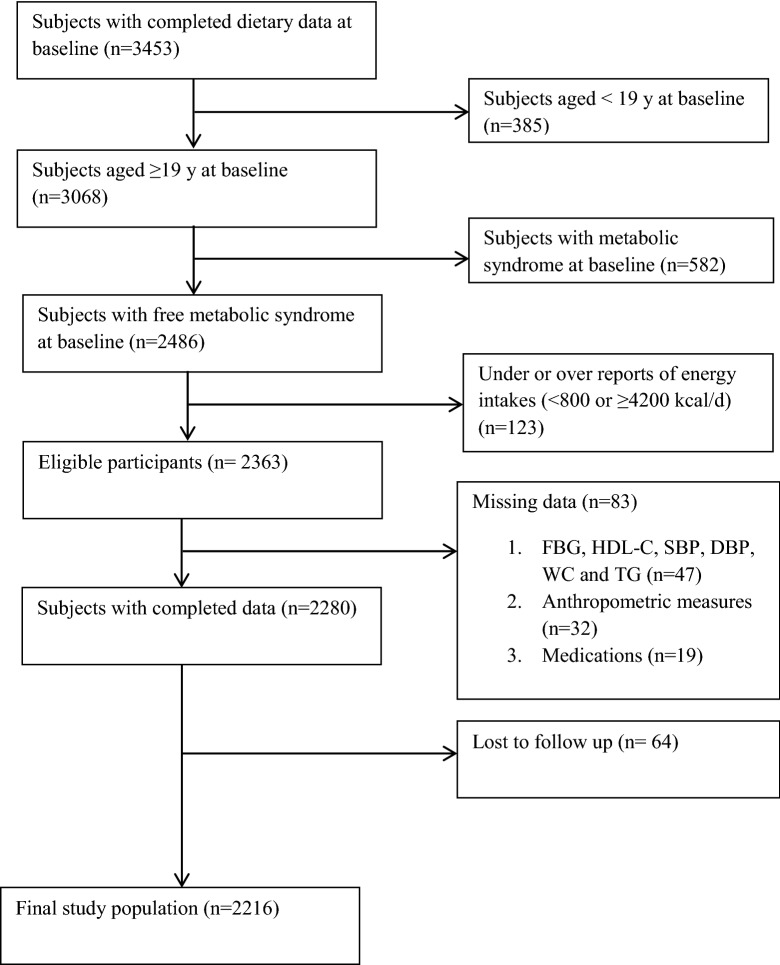
Flowchart for the selection of study participants. *FBG* fasting blood glucose, *HDL-C* high density lipoprotein cholesterol, *WC* waist circumference, *SBP* systolic blood pressure, *DBP* diastolic blood pressure

The current study was conducted within the framework of the Tehran Lipid and Glucose Study (TLGS). Details of the TLGS have been described elsewhere [19]. Briefly, it is an ongoing community-based, prospective study which aims to investigate and prevent non-communicable diseases, through promoting healthy lifestyle. In the third survey of the TLGS (2006–2008), a total of 12,523 subjects aged ≥ 3 years, completed the examinations; of which, 4920 were randomly selected for completing the dietary assessment, based on their age and sex. The dietary data for 3453 subjects who agreed to participate, and completed the food frequency questionnaire (FFQ) were available. The characteristics of individuals who completed the FFQ were similar to those of the total population in the third phase of TLGS [20]. We included subjects who have dietary energy intake between 800 and 4200 kcal/days [21]. After 6.2 years of follow up, according to exclusion criteria, 2216 adults (1064 men and 1152 women), aged ≥ 19 years, were included in the final analyses (Fig. 1).

Written informed consents were obtained from all participants, and the study protocol was approved by the ethics committee of the Research Institute for Endocrine Science, Shahid Beheshti University of Medical Sciences.

### Biochemical measurements

At baseline and follow-up, after 12–14 h of overnight fasting, blood samples were taken into vacutainer tubes, from all participants while in the sitting position. Enzymatic colorimetric method using glucose oxidase, was applied to measure fasting blood glucose (FBG) levels. Serum triglyceride (TG) concentrations were measured through enzymatic colorimetric analysis with glycerol phosphate oxidase, using triglyceride kits. High density lipoprotein cholesterol (HDL-C) concentrations were assayed after precipitation of the apolipoprotein B containing lipoprotein with phosphotungstic acid. All blood analyses were performed at the TLGS research laboratory, using pars Azmoon kits (pars Azmoon inc., Tehran, Iran) and a selectra 2 auto-analyzer (Vital Scientific, Spankeren, Netherlands). Intra- and inter-assay coefficient of variations of all assays were < 5%.

### Measurements

Face-to-face private interviews were performed by qualified interviewers to complete pretested questionnaires. Initially, age, educational level, demographic data, medical history, medication use and smoking habits of individuals were obtained. Weight and height were measured according to the standard method. A digital scale was used to measure weight (nearest 0.1 kg) while individuals were wearing light clothing without shoes. Height of participants was measured to the nearest 0.5 cm, using a stadiometer, while standing with shoulders in normal alignment and without shoes [19]. WC was measured with an accuracy of 0.1 cm, at the umbilical site, in standing position, using non-stretchable tape.

Blood pressure was obtained using a standardized mercury sphygmomanometer; two measurements of blood pressure were taken from the right arm after the participants remained seated for 15-min; the mean of the two measurements was documented as the participant’s blood pressure.

Information on physical activity level was attained using the Persian translated Modifiable Activity Questionnaire (MAQ) [22]. High reliability and relatively moderate validity were reported for the personal translated MAQ in the Tehranian population. The frequency and amount of time spent per day on light, moderate, hard, and very hard intensity activities over the last year, were documented. Physical activity data was expressed as metabolic equivalent hours per week (METs/h/week).

### Definition of metabolic syndrome

MetS components were defined according to modified national Cholesterol Education Program-Adult Treatment Panel III [23], and considering the population- and country-specific cutoff points of WC for Iranian adults [24]. Participants were recognized to have MetS if they had three or more of the components: (1) hypertriglyceridemia, as serum TG ≥ 150 mg/dl or use of anti-hypertriglyceridemia medications, (2) hyperglycemia, as FBG ≥ 100 mg/dl or use of impaired fasting glucose medications, (3) low HDL-C, as serum HDL-C < 40 mg/dl for men, and < 50 mg/dl for women or drug treatment, (4) abdominal obesity, as WC ≥ 95 cm for both men and women, and (5) hypertension, as blood pressure ≥ 130/85 mmHg or use of anti-hypertensive medications.

### Dietary assessment and empirical dietary inflammatory pattern (EDIP)

Information on the habitual diet of participants over the previous year, was collected at baseline, using a valid and reliable 168-item semi-quantitative FFQ. Current FFQ was assessed for reliability of dietary energy and nutrient consumption using a semi-quantitative FFQ (FFQ1 and FFQ2) that was collected with a 14-month interval. For validation of FFQ, a 24-h dietary recall was gathered with a 1-year interval [35]. During face-to-face interview, trained dieticians asked participants to report their frequency of consuming each food item on a daily, weekly, or monthly basis; portion sizes of food intakes reported in household measurement were then converted to grams [20] and serving size. Energy and nutrient content of foods and beverages were analyzed using the US department of agriculture food composition table (FCT). The Iranian FCT was used for some products that were not listed in the USDA FCT.

Dietary data from FFQ were used to calculate EDIP scores. The process of calculating the EDIP score has been explained elsewhere [12]. Briefly, EDIP components consisted of 18 food parameters. Due to religious reasons, wine and beer (anti-inflammatory groups) are not usual or may probably be underreported in the Iraninan population; high-energy and low energy beverages (pro-inflammatory groups) were listed as one food item in the FFQ. Hence for calculating EDIP score, 15 food parameters in two groups were used, based on their inflammatory potential: (1) Anti-inflammatory group, including tea, coffee, dark yellow vegetables (carrots, or squash), leafy green vegetables (cabbage, spinach, or lettuce), snacks (cracker, or potato chips), fruit juice (apple juice, cantaloupe juice, orange juice, or other fruit juice), pizza, (2) Pro-inflammatory group, including processed meat (sausage), red meat (beef, or lamb), organ meat (beef, calf, or chicken liver), other fish (canned tuna, or fish), other vegetables (mixed vegetables, green pepper, cooked mushroom, eggplant, zucchini, or cucumber), refined grains (white bread, biscuit, white rice, pasta, or vermicelli), high-energy and low energy beverages (cola with sugar, carbonated beverages with sugar, fruit punch drinks), and tomatoes. Mean daily intakes of each food group (serving size) weighted by the proposed regression coefficients. To create EDIP, the weighted food group intakes was summed and then divided by 1000 for decreasing the degree of the score and its simplicity to interpret. EDIP scores were recorded as pro-inflammatory diets with more positive scores and anti-inflammatory diets with more negative scores.

### Statistical analysis

The mean ± SD values and frequency (%) of baseline characteristics of the study population were compared across quartile of EDIP score, and also between participants with MetS and without MetS, using the Student’s t test, analysis of variance (ANOVA) and Chi square test, respectively.

Odds ratio (OR) and 95% confidence intervals (CI) of MetS incidence and its components across quartiles of EDIP score, were estimated using logistic regression analysis. Three models were constructed: Crude model with no adjustment, model 1 adjusted for age and sex, model 2 further adjusted for baseline smoking (current smoker, nonsmoker), physical activity (light, moderate, heavy), energy intake (continues), body mass index (BMI) (nonobese < 30, obese ≥ 30), and education (primary and secondary, high school, university) of participants. Tests for trend across EDIP score were performed by assigning the median value of each quartile to the respective categories, and entering this as a continuous variable into the models. Data were analyzed using SPSS (Version 20.0; Inc., IBM Corp., Armonk, NY, USA), with a two-tailed P-value < 0.05 being considered as significant.

## Results

At baseline, the mean ± SD age and BMI of participants were 40.4 ± 12.6 years and 25.9 ± 4.1 kg/m^2^, respectively. The range of EDIP score at the beginning of study was from—2.3 to 6.9. Among the study population, 389 new cases of MetS developed over 6 years of follow-up (incidence rate of MetS was 17.55%). The Mean ± SD of EDIP score was 0.61 ± 0.40. Characteristics of the study participants, according to quartiles of EDIP score, are shown in Table 1. Compared to the highest quartile, participants in the lowest quartile category of EDIP had fewer number of men, lower age and SBP, higher prevalence of current smokers and higher HDL-C (P < 0.05). There was no significant difference between physical activity level, BMI, WC, DBP, FBG, and TG in the highest compared to the lowest quartile of EDIP score.

**Table 1:** Characteristics of the study population across quartile of EDIP score

Characteristics	EDIP score				
	Q1 (n = 554) (< 0.36)	Q2 (n = 565) (0.36–0.55)	Q3 (n = 549) (0.55–81)	Q4 (n = 548) (0.81–6.9)	P
Age (years)	38.9 ± 11.6	36.6 ± 13.2	39.7 ± 12.3	41.3 ± 13.1	0.001
Men (%)	37.9	43.0	58.8	52.6	0.001
Current smoker (%)	10.3	9.7	11.2	9.0	0.004
Physical activity (MET/h/week)	19.1 ± 29.7	20.7 ± 31.4	19.8 ± 31.6	20.5 ± 30.9	0.852
BMI (kg/m^2^)	25.8 ± 4.1	26.2 ± 4.1	26.1 ± 4.2	25.7 ± 3.8	0.416
WC (cm)	88.1 ± 10.2	89.2 ± 10.5	89.2 ± 10.4	98.4 ± 10.1	0.195
SBP (mmHg)	107 ± 11.4	108 ± 12.2	108 ± 12.1	110 ± 11.6	0.001
DBP (mmHg)	72.3 ± 8.5	72.1 ± 8.9	72.8 ± 8.7	72.8 ± 8.5	0.279
FBG (mg/dl)	91.5 ± 13.9	92.4 ± 11.5	92.4 ± 14.8	92.5 ± 9.2	0.526
HDL-C (mg/dl)	51.1 ± 12.2	50.2 ± 11.4	49.2 ± 10.9	49.0 ± 10.6	0.008
TG (mg/dl)	110 ± 58.7	115 ± 58.8	109 ± 52.1	116 ± 64.7	0.161
EDIP score	0.21 ± 0.15	0.45 ± 0.05	0.67 ± 0.07	1.13 ± 0.41	0.001

Data are mean ± SD unless otherwise states (analysis of variance and Chi square test was used for continuous and dichotomous variables, respectively)

EDIP scores were recorded as pro-inflammatory diets with more positive scores and anti-inflammatory diets with more negative scores

*Q* Quartile, *BMI* body mass index, *WC* waist circumference, *SBP* systolic blood pressure, *DBP* diastolic blood pressure, *FBG* fasting blood glucose, *HDL-C* high density lipoprotein cholesterol, *TG* Triglyceride, *EDIP* empirical dietary inflammatory pattern

The dietary intakes of participants by quartiles of EDIP score are illustrated in Table 2. Participants who had a more pro-inflammatory diet had significantly higher intakes of total energy, carbohydrate, fiber, processed meat, red meat, organ meat, other fish, other vegetables, refined grains, tomatoes and beverages (P < 0.05). Participants who had low anti-inflammatory diet had significantly higher intakes of total fat, saturated fatty acids mono-unsaturated fatty acids, tea, and pizza (P < 0.05). Dietary intake of protein, polyunsaturated fatty acids, coffee, dark yellow vegetables, leafy green vegetables, snacks, and fruit juices did not differ by quartiles of EDIP score.

**Table 2:** Nutrients and food group intake of the study population across quartile of EDIP score

Nutrients and food groups	EDIP score				
	Q1 (n = 554) (< 0.36)	Q2 (n = 565) (0.36–0.55)	Q3 (n = 549) (0.55–81)	Q4 (n = 548) (0.81–6.9)	P
Total energy (kcal/day)	1978 ± 647	2196 ± 585	2479 ± 638	2847 ± 643	0.001
Carbohydrate (% of energy)	58.1 ± 7.1	58.6 ± 6.7	58.7 ± 6.1	60.0 ± 5.9	0.001
Protein (% of energy)	14.7 ± 3.1	14.9 ± 4.1	15.2 ± 3.2	14.7 ± 2.4	0.071
Total fat (% of energy)	30.8 ± 6.7	30.3 ± 6.4	29.7 ± 5.5	28.4 ± 5.6	0.001
Saturated fat (% of energy)	10.4 ± 2.9	10.1 ± 2.8	9.8 ± 2.4	9.2 ± 2.4	0.001
Monounsaturated fat (% of energy)	10.3 ± 3.3	10.1 ± 2.6	9.9 ± 2.5	9.4 ± 2.5	0.001
Polyunsaturated fat (% of energy)	6.0 ± 2.1	5.9 ± 1.9	5.9 ± 1.7	5.8 ± 1.8	0.412
Fiber (g/1000 kcal)	16.9 ± 7.6	18.3 ± 5.3	20.2 ± 6.2	22.5 ± 7.9	0.001
Food groups (servings/day)					
Tea	2.9 ± 2.3	2.3 ± 2.4	2.3 ± 1.8	2.1 ± 1.6	0.001
Coffee	0.06 ± 0.3	0.05 ± 0.1	0.04 ± 0.1	0.04 ± 0.1	0.297
Dark yellow vegetables	0.12 ± 0.2	0.13 ± 0.2	0.14 ± 0.2	0.14 ± 0.2	0.186
Leafy green vegetables	0.19 ± 0.62	0.18 ± 0.17	0.19 ± 0.18	0.19 ± 0.18	0.895
Snacks	0.29 ± 0.60	0.23 ± 0.40	0.25 ± 0.39	0.25 ± 0.40	0.158
Fruit juice	0.12 ± 0.25	0.11 ± 0.16	0.11 ± 0.24	0.11 ± 0.25	0.587
Pizza	0.06 ± 0.09	0.04 ± 0.05	0.04 ± 0.04	0.04 ± 0.05	0.001
Processed meat	0.15 ± 0.21	0.17 ± 0.20	0.22 ± 0.34	0.23 ± 033	0.001
Red meat	0.39 ± 0.32	0.50 ± 0.41	0.61 ± 0.52	0.77 ± 0.88	0.001
Organ meat	0.02 ± 0.03	0.02 ± 0.05	0.03 ± 0.05	0.04 ± 0.08	0.001
Other fish	0.17 ± 0.16	0.23 ± 0.20	0.28 ± 0.25	0.40 ± 0.68	0.001
Other vegetables	0.54 ± 0.35	0.70 ± 0.45	0.86 ± 0.65	1.08 ± 2.2	0.001
Refined grains	2.5 ± 1.2	3.8 ± 1.6	5.5 ± 2.1	9.6 ± 4.1	0.001
Tomatoes	0.47 ± 0.36	0.68 ± 0.47	0.80 ± 0.56	0.95 ± 0.92	0.001
Beverages	0.13 ± 0.19	0.17 ± 0.26	0.20 ± 0.28	0.28 ± 0.44	0.001

EDIP scores were recorded as pro-inflammatory diets with more positive scores and anti-inflammatory diets with more negative scores

*EDIP* empirical dietary inflammatory pattern. Data are mean ± SD using Analysis of variance

The association between EDIP score and the risk of MetS and its components are shown in Table 3. Participants with the highest EDIP scores, had higher risk of MetS incidence compared to participants with the lowest score (OR: 2.17, 95% CI 1.56–3.01, P_trend_ = 0.001). After adjusting for potential confounders, this finding remained significant (OR: 1.75, 95% CI 1.21–2.54, P_trend_ = 0.003). Of MetS components, high FBG (OR: 1.46, 95% CI 1.03–2.08, P_trend_ = 0.026), high WC (OR: 1.43, 95% CI 1.03–1.97, P_trend_ = 0.046) and low HDL-C (OR: 1.57, 95% CI 1.34–2.19, P_trend_ = 0.015) had a significant positive association with EDIP score. There were no significant associations between EDIP score, hypertension and high TG.

**Table 3:** Risk of MetS and its components across quartiles of EDIP score

	EDIP score				
	Q1 (n = 554)	Q2 (n = 565)	Q3 (n = 549)	Q4 (n = 548)	P for trend
MetS					
Crude	Ref.	1.40 (0.99–1.98)	1.92 (1.38–2.68)	2.17 (1.56–3.01)	0.001
Model 1	Ref.	1.32 (0.93–1.88)	1.55 (1.10–2.18)	1.88 (1.34–2.62)	0.001
Model 2	Ref.	1.29 (0.90–1.84)	1.47 (1.03–2.09)	1.75 (1.21–2.54)	0.003
High FBG					
Crude	Ref.	1.19 (0.86–1.64)	1.23 (0.89–1.70)	1.54 (1.12–2.10)	0.006
Model 1	Ref.	1.11 (0.80–1.84)	1.22 (0.88–1.69)	1.45 (1.05–1.98)	0.014
Model 2	Ref.	1.11 (0.80–1.55)	1.22 (0.87–1.71)	1.46 (1.03–2.08)	0.026
High WC					
Crude	Ref.	1.37 (1.05–1.78)	1.60 (1.23–2.08)	1.74 (1.33–2.25)	0.001
Model 1	Ref.	1.29 (0.99–1.69)	1.39 (1.06–1.82)	1.53 (1.17–2.01)	0.002
Model 2	Ref.	1.27 (0.94–1.71)	1.29 (0.95–1.76)	1.43 (1.03–1.97)	0.046
High blood pressure					
Crude	Ref.	1.28 (0.92–1.78)	1.30 (0.93–1.80)	1.54 (1.11–2.12)	0.012
Model 1	Ref.	1.14 (0.82–1.60)	1.15 (0.81–1.61)	1.31 (0.94–1.82)	0.117
Model 2	Ref.	1.14 (0.81–1.60)	1.14 (0.80–1.62)	1.33 (0.92–1.92)	0.134
Low HDL-C					
Crude	Ref.	1.23 (0.96–1.59)	1.51 (1.18–1.93)	2.02 (1.58–2.59)	0.001
Model 1	Ref.	1.25 (0.92–1.70)	0.97 (0.72–1.31)	1.86 (1.38–2.51)	0.001
Model 2	Ref.	1.20 (0.88–1.64)	0.88 (0.65–1.20)	1.57 (1.34–2.19)	0.015
High TG					
Crude	Ref.	1.06 (0.79–1.43)	1.58 (1.19–2.09)	1.34 (1.01–1.78)	0.014
Model 1	Ref.	1.02 (0.76–1.38)	1.37 (1.03–1.83)	1.20 (0.89–1.60)	0.117
Model 2	Ref.	1.02 (0.75–1.37)	1.35 (1.01–1.82)	1.16 (0.84–1.60)	0.241

Logistic regression models with 95% confidence interval were used

Crude: no adjustment. Model 1 adjusted for age and sex. Model 2 adjusted further for baseline smoking, physical activity, energy intake, BMI, and education of participants

*Q* Quartile, *MetS* metabolic syndrome, *EDIP* empirical dietary inflammatory pattern, *WC* waist circumference, *SBP* systolic blood pressure, *DBP* diastolic blood pressure, *FBG* fasting blood glucose, *HDL-C* high density lipoprotein cholesterol, *TG* triglyceride

## Discussion

In the current study, EDIP was used to evaluate the inflammatory potential of diet on a group of adult TLGS participants. Our study is the first to assess the association between EDIP and MetS in a Middle Eastern country. Higher values of EDIP indicated a higher pro-inflammatory diet. This prospective study showed a positive association between EDIP score and risk of MetS incidence. We also assessed the association of EDIP score with MetS components; participants with higher EDIP score had a higher risk of high FBG, high WC, and low HDL-C; however, no association was found between EDIP score, hypertension and hypertriglyceridemia.

The association between EDIP score and the risk of MetS incidence is consistent with Neufcourt et al. study; the odds of MetS in participants with higher pro-inflammatory diet was 39% higher compared to those with the higher anti-inflammatory diet [16]. In contrast with our results, in two cross-sectional and one cohort studies, no significant association was found between DII score and the risk of MetS incidence [13–15]. Pimenta et al. [15] reported that in participants from the SUN cohort study, there was no significant association between DII score and MetS, during a mean 8.3 years of follow-up. This controversy may be induced from differences in type of studies, or differences in calculation of DII [13, 14]. In the current study, a hypothesis-derived empirical index that has been proposed by Tabung et al. was used to evaluate the inflammatory potential of diet. Using this dietary inflammatory index refers to the inflammatory effects of foods in whole diets. In addition, compared to nutrient-food parameters, using the inflammatory index based on food groups, facilitates providing nutritional recommendations to reduce levels of diet-induced inflammation [12].

Previous studies assessed the association between diet quality and inflammatory markers [25, 26]. Several studies have shown that fruit and vegetable intakes are inversely associated with inflammatory biomarkers, especially C-reactive protein (CRP) [27, 28]. While the western dietary pattern, high in red meats and fast foods, is positively associated with high inflammatory biomarkers, particularly CRP and interleukin-6 (IL-6) [29]. It has been hypothesized that increase in pro-inflammatory cytokines and reduction of anti-inflammatory cytokines induced by the high pro-inflammatory diet, leads to an increased risk of chronic inflammation and MetS [30]. The Mediterranean dietary pattern that emphasizes on high consumption of fruit and vegetables, and low consumption of meat and meat products, has shown to have an anti-inflammatory potential [29, 31, 32]. Furthermore, previous studies found that adopting the Mediterranean dietary pattern has a preventing effect on MetS, also having a negative association with serum glucose and TG concentration, and positive association with serum HDL-C concentration [33, 34].

In our study, a high risk of abdominal obesity was observed in participants with high EDIP score. This result confirms the hypothesis of Ruiz-canela et al. study, that high pro-inflammatory diet might increase the risk of obesity. In this investigation from the PREDIMED study, DII score was positively associated with WC [17].

Our results are in line with data from the Polish-Norwegian study (PONS), a cross-sectional study on 3862 participants, which reported an inverse association between DII score and serum HDL-C, and a positive association with serum TGs [13]. Similarly, Neufcourt et al. showed that a higher pro-inflammatory diet is associated with higher TGs, and lower HDL-C [16]. In another study on 90 participants with excess body weight (26 ≤ BMI ≤ 35), there was an inverse association between anti-inflammatory diet and serum glucose and TG [30], which was consistent with our findings.

Our study has some limitations. First, some factors such as heredity may need to be mentioned, because of heterogeneity of the MetS. In addition, EDIP score was determined based on 16 out of the 18 food parameters of the original EDIP; the dietary components, wine and beer, were not included due to religious reasons. The Use of the USDA’s food composition table was another limitation of current study. Finally, lack of data on blood levels of inflammation biomarkers, was another limitation of this study to develop our own population coefficients.

However, the current study also has some strengths. First, our study was conducted in a Middle Eastern country, with a population-based prospective design, large sample size and long follow-up. Furthermore, comprehensive measurement of dietary consumption using a valid and reliable FFQ is another strength of this study [35], which provided an accurate estimation of EDIP. Also, a validated physical activity questionnaire was used to estimate energy expended for physical activity.

## Conclusion

In this prospective, population-based study with 6.2 years of follow-up, higher EDIP score that indicated a more pro-inflammatory diet was associated with an increased risk of MetS, hyperglycemia, low HDL-C and high WC in adults.
